# Supplementary material for: Using a computational cognitive model to simulate the effects of personal and social network experiences on seasonal influenza vaccination decisions
Source: Front Epidemiol. 2024 Nov 13;4:1467301. doi: 10.3389/fepid.2024.1467301 (PMC11603355; doi:10.3389/fepid.2024.1467301)
Supplement: Supplementary file 1 [file Datasheet1.docx]

**Supplementary Materials**

**Survey Questions**

The first wave of the survey was administered in Fall 2015 (Supplementary Table 1). The survey included two questions asking participants to retrospectively report the number of years since they last got the influenza vaccine and the number of years since they last had an illness that they thought was the flu. We coded these responses to reflect the participants’ vaccination and illness experiences from the previous season. The first wave of the survey also asked participants to identify up to 15 close social contacts. They were given the prompt, “From time to time, most people discuss important matters with other people. Looking back over the last several years, who are the people with whom you discussed matters important to you? Please list up to 15 of these people individually. Please only consider people who are 18 years old or older.” Finally, for each social contact identified, participants were asked to state how strongly they suspected that they had caught the flu and got the flu vaccine. Participants chose from five response levels ranging from “Definitely yes” to “Definitely no”. We coded responses of “Definitely yes” and “think yes” as *yes*, and we coded responses of “Definitely no” and “think no” as *no*.

The second wave of the survey was administered in the Spring 2016 (Supplementary Table 1). This survey and all later Spring surveys included a sequence of questions asking participants about whether they vaccinated in the previous season in response to a provider recommendation and, if not, whether they vaccinated without a recommendation. The survey also asked participants whether they had an illness they thought was influenza during the previous season. Finally, participants were asked how strongly they suspected that each of the social contacts they listed had caught influenza and got the influenza vaccination. These responses were coded in the same was as for the first wave of the survey.

To summarize, a total of eight surveys were administered over four years. Because the first survey included retrospective questions about the previous influenza season, data from the surveys covered a total of five years.

*Supplementary Table 1. Vaccination and Influenza Questions Included on Survey*

| Season | Question |
| --- | --- |
| Fall 2015 | 1. (Ego) How long ago did you last think you had the flu? 2. (Ego) How long ago did you last get the seasonal flu vaccine? 3. (Alter) For each of the people on the list, indicate if you know or strongly suspect that they caught the flu during the last flu season (Fall 2015 to Spring 2016). 4. (Alter) For each of the people on the list, indicate if you know or strongly suspect that they got the flu vaccine during the last flu season (Fall 2015 to Spring 2016). |
| Spring 2016, Fall 2016 to Spring 2020 | 1. (Ego) Since August 20xx, has a healthcare provider personally recommended that you get vaccinated for the flu, either in person or by phone or email? 2. (Ego) Did you receive the flu vaccine in response to this recommendation? 3. (Ego) Have you been vaccinated for the flu this year (since August 20xx)? 4. (Ego) Since August 2018, have you had an illness that you think was the flu? 5. (Alter) For each of the people on the list, indicate if you know or strongly suspect that they caught the flu this year. 6. (Alter) For each of the people on the list, indicate if you know or strongly suspect that they got the flu vaccination this year. |
